# Factors Influencing the Acceptance of Pediatric Telemedicine Services in China: A Cross-Sectional Study

**DOI:** 10.3389/fped.2021.745687

**Published:** 2021-10-18

**Authors:** Jingjin Shi, Xueming Yan, Miao Wang, Ping Lei, Guangjun Yu

**Affiliations:** ^1^School of Public Health, Shanghai Jiao Tong University School of Medicine, Shanghai, China; ^2^International Peace Maternity and Child Health Hospital, School of Medicine, Shanghai Jiao Tong University, Shanghai, China; ^3^Shanghai Children's Hospital, Shanghai Jiao Tong University, Shanghai, China; ^4^Institut Franco-Chinois pour la Santé, Chambéry, France

**Keywords:** telemedicine, acceptance, influencing factor, pediatric hospital, China

## Abstract

**Background:** Pediatrician workforce shortages have aroused great attention from health authorities in China. Telemedicine services have been known to enhance the management of children's health, yet the rate of adoption and usage in Chinese hospitals still at a quite low level, and the factors influencing the acceptance of telemedicine services remains unclear.

**Objective:** The purpose of this empirical study was to evaluate the reliability and validity of a technology acceptance measurement instrument applied in healthcare, to investigate the perception of telemedicine services on the provider-side and demand-side, and to determine the factors that may drive individuals to adopt telemedicine services.

**Methods:** A cross-sectional survey study based at Shanghai Children's Hospital, Shanghai Jiao Tong University, was conducted in March 2020. A total of 456 valid responses were obtained by convenience sampling. The internal consistency of items was assessed by Cronbach's alpha (α), composite reliability (CR) and average variance extracted (AVE) to evaluate both the reliability and validity of the questionnaire. Structural equation modeling analysis was used to test and verify the interrelationships among relevant variables.

**Results:** Price value is the strongest predictor (β = 0.30, *p* = 0.02), facilitating conditions (β = 0.28, *p* = 0.01) and hedonic motivation (β = 0.13, *p* = 0.04) also have significantly positive direct effects on telemedicine acceptance. The results showed the perception of child patients' families were significantly more acceptable to telemedicine services than pediatricians (*t* = −2.99, *p* < 0.01). Participants with no prior experience and lower education may be more willing to adopt telemedicine.

**Conclusion:** Telemedicine will likely continue to have an integral role in pediatric health care delivery, and the findings can assist policy makers and hospital administrators in determining the more valued characteristics of telemedicine services from a behavioral perspective. Future attention will be paid to the pricing, training and service quality of telemedicine in China.

## Introduction

Telemedicine refers to the use of health information exchanged from one site to another via information and communications technology (ICT) for the health and education of the patient or medical personnel with the intention of evaluating, diagnosing, treating, educating or managing patients (1, 2). With the rapid development of ICT, telemedicine has been used widely around the world as a new mode of medical service (3). As the largest developing country with a large population, medical resources in China are distributed with imbalanced and uneven quality, especially in pediatric medical resources (4). Facing a serious situation of high demand for medical resources and shortage of pediatricians, telemedicine has been considered a crucial solution by Chinese health authorities to alleviate healthcare disparities and to improve the accessibility, affordability and quality of medical resources in urban and rural areas. To date, telemedicine has a history of more than 30 years in China since it began to develop in the 1980s. The outbreak of the COVID-19 epidemic has enhanced and accelerated the worldwide development of telemedicine, which has helped reduce the chances of cross-infection and overcome the geographical limitations of medical treatment (5). It is disappointing that the adoption of telemedicine has not been consistent with its technological advancements (6). There has been a growing need, but few studies have explored factors affecting the willingness to use telemedicine services in pediatric hospitals. To achieve this goal, the measurement instrument based on the unified theory of the acceptance and use of technology 2 (UTAUT 2), which is recognized as the most comprehensive theory in measuring individual technology acceptance (7, 8), was employed to investigate the interrelationships between the constructs and behavioral intention and to estimate the significance of path coefficients so that we could better understand the factors that may influence the willingness to accept telemedicine services.

### Theoretical Background and Hypothesis Development

**Figure 1:**
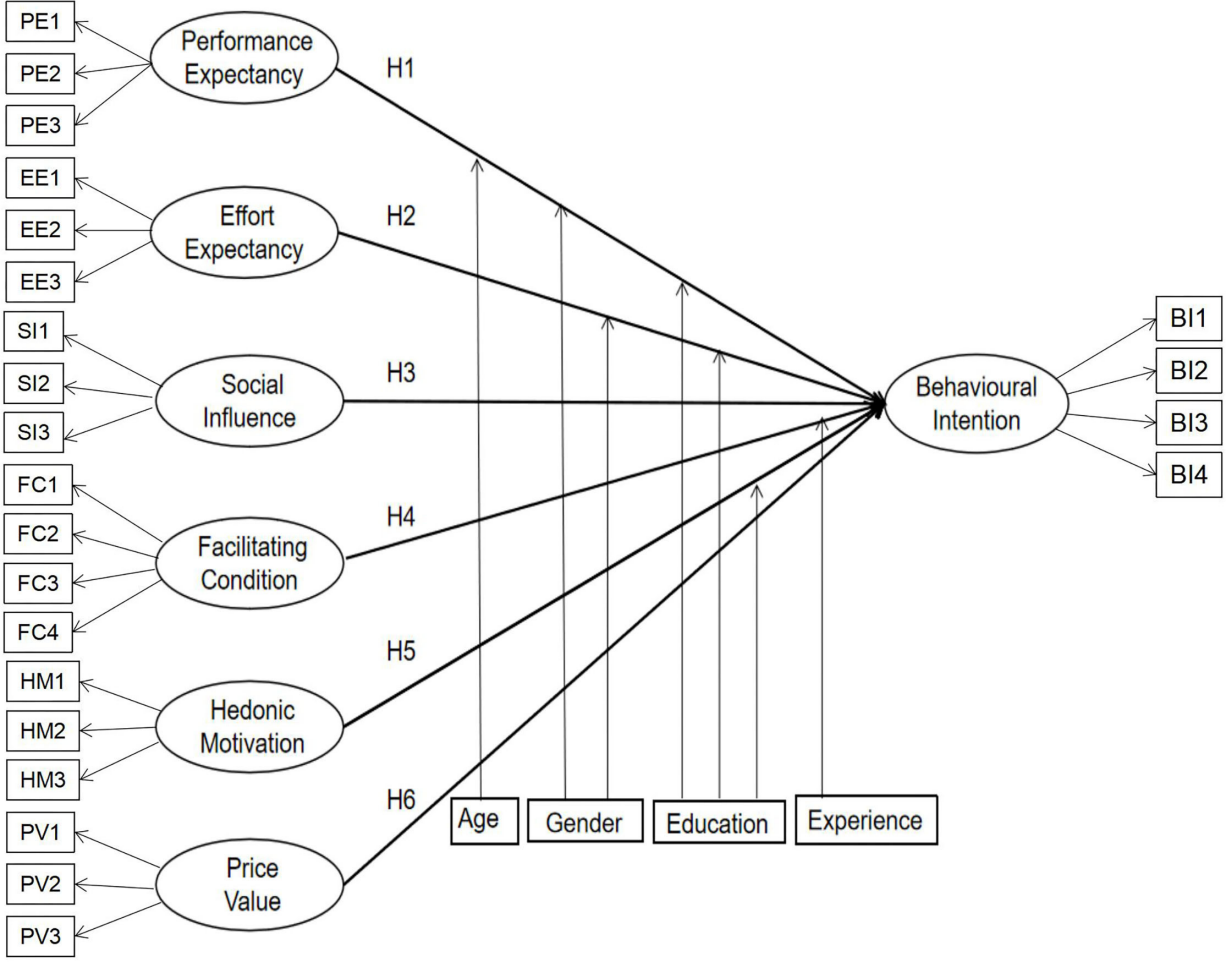
Research framework.

UTAUT 2 provides a more thorough understanding of the factors that influence users' willingness to use new technology than is possible with other technology acceptance models. A systematic review has shown that UTAUT 2 is an efficient theory, with the minimum explained variance of behavioral intention being 35% and the maximum value being 94% (9). Although the UTAUT 2 model was not specifically developed for healthcare, it is perceived to be a robust integrative theory focused on medical providers and users (10). Through induction and summary, we have defined the model variables; the measurement items and model construction of each research variable in the model have also been proposed, as seen in Table 1. The hypothesized research model is depicted in Figure 1.

**Table 1:** Construct definitions and model assumptions.

**Model variable**	**Definition**	**Model assumptions**	**References**
Performance expectancy	Refers to the degree to which an individual believes that using a specific technological application will help him or her to improve job performance	Performance expectancy has a positive direct effect on hospital users' willingness to use telemedicine services	(11, 12)
Effort expectancy	Refers to the degree of simplicity associated with an individual's perception of a given system	Effort expectancy has a positive direct effect on hospital users' willingness to use telemedicine services	(13)
Social influence	Refers to how an individual perceives that “important others” view them in affecting whether they should use the technology	Social influence has a positive direct effect on hospital users' willingness to use telemedicine services	(14, 15)
Facilitating conditions	Refers to the degree to which an individual believes that an organizational and technical infrastructure supports the implementation of a technology	Facilitating conditions have a positive direct effect on hospital users' willingness to use telemedicine services	(16–18)
Hedonic motivation	Refers to the fun or pleasure derived from using a technology	Hedonic motivation has a positive direct effect on hospital users' willingness to use telemedicine services	(19)
Price value	Refers to the cognitive tradeoff between the perceived benefits of a given system and the monetary cost of using them	Price value has a positive direct effect on hospital users' willingness to use telemedicine services	(20–22)

## Methods

### Study Design and Sampling

This is a cross-sectional study investigating hospital users' willingness to use telemedicine services. At present, telemedicine service centers are mainly situated in the most developed cities in China, such as Beijing, Shanghai, and Guangzhou (23). Similar facilities are not available in many less developed cities and regions. This research setting was a tertiary pediatric specialized hospital in Shanghai, a mega-city with the largest population (nearly 25 million) in China. This survey was initiated in March and concluded in May 2020 at Shanghai Children's Hospital, which is the first established specialized children hospital in China. The target population are the end-users of telemedicine system, such as pediatricians and family members of pediatric patients. Family member refers to the parent or guardian of the child patient. Considering the low awareness and utilization rate of telemedicine services, the target group participants filled out the electronic questionnaire by scanning the code on their mobile phones through posted flyers on the nurse stations of Shanghai Children Hospital. The electronic questionnaires were answered anonymously and were collected on the spot. The inclusion criteria for participants included the following: (1) ability to fill out the questionnaire independently, with clear consciousness and no obvious cognitive impairment; (2) willingness to voluntarily participate in this study; and (3) age range of 18–60 years. The exclusion criteria included the following: (1) having a mental disorder that prevented normal communication and (2) refusing to participate in this investigation. Prior to the survey, trained investigators explained telemedicine services to the respondents to assist them in understanding the meaning of the survey questions. A sample of 456 valid questionnaires was eventually collected. This study was approved by the Ethics Review Committee, Shanghai Children's Hospital, Shanghai Jiao Tong University (file number 2021R077-E01).

### Measurements

All of the items were based on the UTAUT 2 model measuring the acceptance of new information technologies, which was adopted from Venkatesh et al. (18) and Gao et al. (22) with necessary validation and wording changes tailored to the telemedicine service and healthcare context. The items are shown in Supplementary Material. The questionnaire was administered in Chinese through a web hosting service after being translated by a professional translator. To ensure that the content did not lose its original meaning, a back-translation was made from the Chinese instrument to English, again by a professional translator, and compared to the original (24). All questions were measured using a 7-point Likert scale ranging from 1 (completely disagree) to 7 (completely agree). A draft set of survey questions was refined by employing cognitive interviews and a pretest. Interviewees (*n* = 5) who were postgraduates majoring in health informatics or end users of telemedicine services were asked to verbalize the mental process entailed in providing answers. The wording of the questions that were difficult to understand or that generated ambiguity was subsequently modified based on the feedback from 50 respondents in the pretest.

### Statistical Analysis

Once the primary collection was completed through a structured electronic questionnaire, the data were coded, cleaned, labeled, and verified with regard to missing values. A two-step approach was employed for structural equation modeling (25). The reliability and validity of the measurement model was examined in the first step, and the structural model was tested in step two. Confirmatory factor analysis was performed to validate the measurement model, and the hypothesized paths were examined using structural equation modeling. The moderating effects of three end-user traits among the hypothesized paths within the core research model were tested using logistic regression analysis and multiple group analysis. Additionally, independent *t*-tests were conducted to test differences in perception and intention to use between physicians and family members of patients. According to the degree of consistency between the theoretical model and the actual data, the theoretical model is evaluated to achieve the goals of quantitative research on actual problems. Structural equation modeling overcomes the shortcomings of multiple regression analysis method. It not only explains the relationship between variables but also allows the existence of measurement error of the variables. It can realize the estimation of factor structure and relationship as well as the simultaneous estimation of the degree of model fitting. All analyses were conducted using SPSS Statistics, version 24.0 (IBM Corp), and SPSS Amos, version 24.0 (IBM Corp) software.

## Results

### Study Population

In this study, a total of 456 respondents completed the survey, including physicians (53.5%) and family members of patients (46.5%). As shown in Table 2, 18% of the respondents were men, and 82% were women. The largest age group was 30–40 years (42.3%). The group with the highest level of education had bachelor's degrees (51.3%).

**Table 2:** Demographics of the respondents (*N* = 456).

**Measure**	**Items**	**Frequency**	**Percentage (%)**
Gender	Male	82	18.0
	Female	374	82.0
Age	20–30	127	27.9
	31–40	193	42.3
	41–50	90	19.7
	51–60	46	10.1
Status	Pediatricians	244	53.5
	Family member of pediatric patients	212	46.5
Level of education	High school or below	72	15.8
	College	89	19.5
	Bachelor	234	51.3
	Master	51	11.2
	Doctor	10	2.2

### Reliability and Validity of Measurement Instrument

The 23-item scale appeared to be internally consistent. The Cronbach α for the 7 subscales ranged from 0.890 to 0.957, indicating that the measurement scale had good reliability (see Supplementary Table 1). Convergent validity was adequate when the factor loading and the composite reliability (CR) were above the recommended threshold of 0.7 and the AVE was greater than 0.5 (26) (see Supplementary Table 2). Discriminant validity was also confirmed when the square root of the AVE for each construct was larger than the corresponding inter-construct correlations (27). Hence, this measurement model achieved acceptable levels of reliability and validity. Additionally, confirmatory factor analysis indicated a good fit, as the fit indices for the measurement model exceeded the critical level of 0.80, and the chi-square/degree of freedom equals to 2.714, which was below the suggested value of 3.0 (28). The indicators were as follows: goodness-of-fit index (GFI) = 0.904, normed fit index (NFI) = 0.957, comparative fit index (CFI) = 0.972, root mean square residual (RMR) = 0.062, root mean square error of approximation (RMSEA) = 0.061. It can be seen that the variables had good discriminant validity. According to the degree of consistency between the theoretical model and the actual data, the theoretical model was evaluated to achieve the goals of quantitative research on actual problems. In sum, our results indicated the appropriateness of the measurement model.

### Perceptions of Telemedicine Services

A two-independent samples *t*-test was carried out focused on physicians and family members of patients in relation to the perception of influencing factors on the acceptance of telemedicine services. Overall, there was a positive perception of telemedicine services in a Chinese pediatric hospital among the participants with all mean scores larger than 5. The findings indicate that family member of pediatric patients reported a more positive and optimistic perception toward telemedicine services vs. pediatricians in all subscales of measurement. Table 3 reported on the means and standard deviations of perception of telemedicine services. The mean scores of facilitating conditions (*t* = −2.19, *p* = 0.03), hedonic motivation (*t* = −2.65, *p* < 0.01), price value (*t* = −3.26, *p* < 0.01) and intention to use telemedicine (*t* = −2.99, *p* < 0.01) were significantly higher on the demand-side than on the provider-side. Respondents were the most positive about the performance expectancy (mean = 6.09, SD = 1.08). However, respondents reported the lowest mean score (mean = 5.24, SD = 1.48) on the hedonic motivation.

**Table 3:** Perception of telemedicine services by pediatricians and family members of patients.

**UTAUT 2 constructs**	**All**	**Pediatricians**	**Family member of pediatric patients**	***t*-test**	***P*-value**
	**Mean (SD)**	**Mean (SD)**	**Mean (SD)**		
PE	6.09 (1.08)	6.01 (1.08)	6.19 (1.07)	−1.76	0.08
EE	5.89 (1.25)	5.78 (1.18)	6.01 (1.31)	−1.96	0.05
SI	5.42 (1.50)	5.35 (1.35)	5.49 (1.67)	−1.01	0.31
FC	5.69 (1.31)	5.56 (1.23)	5.83 (1.38)	−2.19	0.03
HM	5.24 (1.48)	5.07 (1.33)	5.44 (1.61)	−2.65	<0.01
PV	5.68 (1.24)	5.51 (1.22)	5.89 (1.25)	−3.26	<0.01
BI	5.51 (1.38)	5.33 (1.28)	5.72 (1.47)	−2.99	<0.01

*PE, performance expectancy; EE, effort expectancy; SI, social influence; FC, facilitating condition; HM, hedonic motivation; PV, price value; BI, behavior intention; SD, standard deviation*.

### Hypothesis Testing

In this study, model verification of the parameters of the initial hypothetical model was carried out to analyse the relationship between the variables and the mechanisms of influence (see Table 4). A *P* value <0.05 was considered statistically significant. To explain the variance of the constructs, the *R*^2^ values were examined. With an *R*^2^ value of 0.737, our model explains 73.7% of the variance in behavioral intention determined by three variables: facilitating conditions (H4: β = 0.280, *p* = 0.008), hedonic motivation (H5: β = 0.131, *p* = 0.040) and price value (H6: β = 0.302, *p* = 0.016). Given the significance of the model path coefficient (β), H4, H5, and H6 are accepted, while the other variables of the model, such as performance expectancy (H1), effort expectancy (H2), and social influence (H3), are rejected. **Table 6** clearly shows that facilitating conditions, hedonic motivation, and price value are the three main factors affecting pediatric hospital users' behavioral intention to adopt telemedicine services. They all had a positive impact on behavioral intention, and among the three factors, price value seems to be the most powerful influencing factor.

**Table 4:** Results of hypothesis testing.

**Hypotheses**	**Path**	**Estimate**	**Standard error**	***P*-value**	**Findings**
H1	PE → BI	0.09	0.07	0.09	n.s.
H2	EE→ BI	0.08	0.08	0.32	n.s.
H3	SI→ BI	0.04	0.07	0.58	n.s.
H4	FC→ BI	0.28	0.11	0.01	Supported
H5	HM→ BI	0.13	0.07	0.04	Supported
H6	PV→ BI	0.30	0.13	0.02	Supported

*PE, performance expectancy; EE, effort expectancy; SI, social influence; FC, facilitating condition; HM, hedonic motivation; PV, price value; BI, behavior intention. n.s., not significant*.

### Moderating Effects of Age, Gender and Experience

First, we conducted a separate test by establishing a logistic regression model to measure the relationship between gender, age, education level, experience, and willingness to use telemedicine service (Table 5). Participants with no prior experience and lower education may be more willing to adopt telemedicine service. Second, we performed an individual estimation for age (younger or older), gender (male or female) and experience (none or experienced), education level (lower or higher) and then conducted a multi-group analysis to determine whether the moderating effects of hypothesized paths were different between sub-groups. The chi-square differences between the unconstrained model and constrained model are presented in Table 6. In the unconstrained model, all paths were unconstrained between the two sub-groups. In the constrained model, each path was constrained as equal and was hypothesized to be moderated across two sub-groups. We found that the path between PE and BI was significantly stronger for males than for females (Δχ^2^ = 3.82, *p* = 0.05) and for older than for younger individuals (Δχ^2^ = 8.63, *p* < 0.001). The moderating effect of age and gender on the relationship between FC and BI was not significant, but the results for individuals who were younger (β = 0.35, *p* = 0.01) and female (β = 0.30, *p* = 0.02) showed a statistically stronger relationship between FC and BI. The moderating effect of experience on the relationship between FC and BI (Δχ^2^ = 6.02, *p* = 0.01) was significant, and the path coefficient for not experienced participants (β = 0.45, *p* < 0.001) was larger than that for those who had already used telemedicine services (β = −0.03, *p* = 0.83). In the age results, although the path from PV to BI was stronger for the younger group, the standardized path coefficient was not statistically significant. In the gender results, the path from EE and HM to BI was stronger for the female group, and the standardized path coefficient was statistically significant. In addition, the multi-group analysis indicated that the difference in chi-square values was statistically significant between EE and BI (Δχ^2^ = 4.67, *p* = 0.03) but not between FC, HM and BI. With respect to the new moderator, the moderating effect of education level on the relationship between PE (Δχ^2^ = 5.24, *p*=0.02), EE (Δχ^2^ = 6.27, *p* = 0.01), and HM (Δχ^2^ = 5.36, *p* = 0.02) toward adopting telemedcine service was significant and participants with lower education showed a stronger relationship.

**Table 5:** The relationship between demographic characteristics and behavior intention to use telemedicine.

**Variable**	***B***	**SE**	**Wald value**	***P*-value**	**Odds ratio (95%CI)**
Gender^a^	−0.08	0.27	0.09	0.76	0.92 (0.55–1.56)
Age	−0.07	0.11	0.43	0.51	0.93 (0.75–1.15)
Experience	−0.74	0.20	13.20	<0.001	0.48 (0.32–0.71)
Education level	−0.25	0.12	4.05	0.04	0.78 (0.62–0.99)

a *Male as the reference group*.

**Table 6:** Results for moderating effects models.

**Moderator**	**Path**	***X*^**2**^(df)**	**Δ*X*^**2**^**	**Standardized path coefficient**	
Age				Younger (<40 years)	Older (>40 years)
	Unconstrained model	932.27(414)			
	Constrained model: PE→ BI	936.09(415)	3.82^*^	0.001	0.24^*^
	Constrained model: EE→ BI	933.27(415)	1.00	0.15	−0.04
	Constrained model: FC→ BI	932.65(415)	0.38	0.35^*^	0.25
	Constrained model: HM→ BI	932.43(415)	0.16	0.09	0.15
	Constrained model: PV→ BI	932.57(415)	0.30	0.36^*^	0.20
Gender				Male	Female
	Unconstrained model	980.11(414)			
	Constrained model: PE→ BI	988.74(415)	8.63^**^	0.40^**^	0.001
	Constrained model: EE→ BI	984.78(415)	4.67^*^	−0.65	0.23^*^
	Constrained model: FC→ BI	980.12(415)	0.01	0.27	0.30^*^
	Constrained model: HM→ BI	980.17(415)	0.06	0.13	0.16^*^
	Constrained model: PV→ BI	980.79(415)	0.68	0.52	0.20
Experience				Experienced	No experience
	Unconstrained model	912.65(414)			
	Constrained model: PE→ BI	912.69(415)	0.05	0.16	0.09
	Constrained model: EE→ BI	912.70(415)	0.06	0.14	0.06
	Constrained model: FC→ BI	918.66(415)	6.02^**^	−0.03	0.45^**^
	Constrained model: HM→ BI	912.74(415)	0.09	0.23	0.10
	Constrained model: PV→ BI	912.75(415)	0.10	0.16	0.18
Education level				Lower	Higher
	Unconstrained model	1104.94(414)			
	Constrained model: PE→ BI	1110.18(415)	5.24^*^	−0.18	0.14^*^
	Constrained model: EE→ BI	1111.21(415)	6.27^**^	0.41^**^	0.001
	Constrained model: FC→ BI	1105.19(415)	0.25	0.33	0.23^*^
	Constrained model: HM→ BI	1110.30(415)	5.36^*^	0.40^**^	0.04
	Constrained model: PV→ BI	1106.29(415)	1.35	0.03	0.42^*^

*PE, performance expectancy; EE, effort expectancy; FC, facilitating condition; HM, hedonic motivation; PV, price value; BI, behavior intention*.

* *p < 0.05*.

** *p < 0.01*.

## Discussion

Telemedicine has the potential to benefit pediatric care by increasing access to pediatric specialists and remotely delivering high-quality health services, including radiology, mental health, dermatology, cardiology, pathology, patient education, chronic diseases, pediatric dentistry, and neonatal ophthalmology (29, 30). In China, the current operational mode of telemedicine is limited in the business-to-business context and for common and chronic diseases (4, 31); therefore, the functions of telemedicine services are not yet fully used. This study aimed to determine which factors influence the acceptance of telemedicine services in a Chinese pediatric specialized hospital.

### Price Value

In our study, price value is the most important influential factor, particularly for younger users, which aligns with previous research on the same topics (32, 33). However, some studies based on the UTAUT 2 model did not include PV as a predictor for the acceptance of a new technology (22, 34). Meanwhile, it is noteworthy that the path coefficient between PV and behavioral intention had some differences among countries. Comparing the path coefficients of 0.130 in the United States (33), 0.147 in France (12), 0.320 in Iran (32), and 0.302 in China, one may conclude that price value may have a greater impact in less developed countries. Payer reimbursement was the leading influencer of anticipated future use of telemedicine (35). To date, telemedicine services have not been covered by medical insurance payments in China, and the prices vary from province to province. However, insurers in the United States have expanded their coverage and reimbursement of various types of telemedicine services. Scholars believe that if the price of certain technological services is very low or free of charge, it probably will not have a strong influence on behavioral intention (4, 36). Physicians and patients are more likely to take advantage of telemedicine if their perception of its value is higher, such as saving them money and time by avoiding an out-of-town trip to the hospital (11). Telemedicine charges and reimbursement standards should be constructed to meet the actual local needs, and the labor value of telemedicine service providers should be reasonably compensated, including material and spiritual, monetary and non-monetary compensation incentive strategies.

### Facilitating Conditions

Facilitating conditions were also a significant predictor of the intention to use telemedicine in this study, which was consistent with previous studies (37). Training can not only increase the confidence of telemedicine users but also strengthen collaboration between patients and physicians. In terms of external conditions, the infrastructure, system interface, image quality, network signal and transmission speed of telemedicine systems all need to be ready and periodically maintained, especially in rural areas. In terms of internal conditions, the service process should be optimized to shorten the waiting time. The content of cooperation, service processes, rights and obligations, risks and responsibilities between medical institutions should be determined in a formal agreement before the launch of telemedicine services. Considering the moderating effects of age, gender and experience, female and young users without experience had more positive behavior intention of adopting telemedicine services. Male and older experienced users need more organizational and technical infrastructure supports for the implementation of telemedicine services.

### Hedonic Motivation

Hedonic motivation also had a positive impact on behavioral intention (38). The adoption of a product based on hedonic motivation is not long term. Once adopters acquire experience with it, its effectiveness outweighs all its other attributes (18). Telemedicine has just taken off in China in recent years. Early adopters pay more attention to its convenience but easily overlook the importance of medical quality. However, telemedicine cannot be merely considered a pleasure-oriented service but is perceived as more of a utilitarian solution (18). Hospitals should provide telemedicine services on the premise of ensuring medical safety and being closely connected with offline medical treatment.

### Performance Expectancy, Effort Expectancy, Social Influence and Moderators

From our results, pediatricians and family members of patients were consistently agreed performance expectancy and effort expectancy as important factors of adopting telemedicine service. Even though these positive effects on behavior intention have not been validated in this study. This may be because users have not yet changed their traditional perceptions and are not fully aware of the usefulness of telemedicine services. Pediatric patients have difficulty operating the system and communicating with physicians due to their limited understanding. In a tertiary hospital in China, clinicians are often constrained by their busy work schedules and therefore have limited energy for learning how to work with new technologies. To solve this problem, hospitals could equip technical assistants responsible for communication and system operation. Individuals could also be impacted by people who are important to them when adopting a new technological product (39). We speculate that healthcare and medical treatment for minors is a very personal and private issue; therefore, SI had limited impact in our study, and previous studies found the same results (40).

In addition, certain attributes may influence users' decisions. In healthcare studies, gender and age were found to be moderators of acceptance (41, 42). Regarding experience, users and non-users showed significant differences regarding PE, EE, SI and FC in the acceptance of mobile health monitoring services (1, 43). The results of moderating effects of age, gender and experience deepen our understanding of underlying differences in telemedicine services adoption behavior. We found that gender moderates the relationships of PE→ BI and EE→ BI. This finding is similar to what Duyck et al. (44) suggested: PE is a stronger factor for males than for females. The relation between EE and BI is stronger for women, which is consistent with previous research (15). Participants with no prior experience and lower education with telemedicine may be more willing to try this new type of technology. The results suggest that hospital administrators should focus more on increasing publicity to enhance user awareness of telemedicine, strengthening homogeneous management of online and offline medical quality, so that acquiring positive feedback from respondents who have used it.

### Limitations and Future Research

The study has some limitations that can be regarded as opportunities for future research. First, this study recruited a cross-sectional sample of participants in Shanghai; it may not universally reflect the willingness of all users to adopt telemedicine services in Chinese pediatric hospitals, especially in rural areas. Further research in different regions will provide more accurate evidence if the results depend on socioeconomic factors. Second, telemedicine involves the privacy protection and information security of personal health data, and future research should incorporate perceived risk and trust factors to further extend the theoretical model. Third, females were over-represented in the respondent groups, and respondents younger than 40 years old accounting for 70% in the context of pediatric hospital. Further studies should collect data following the gender and age ratios of the general population.

## Conclusion

**Figure 2:**
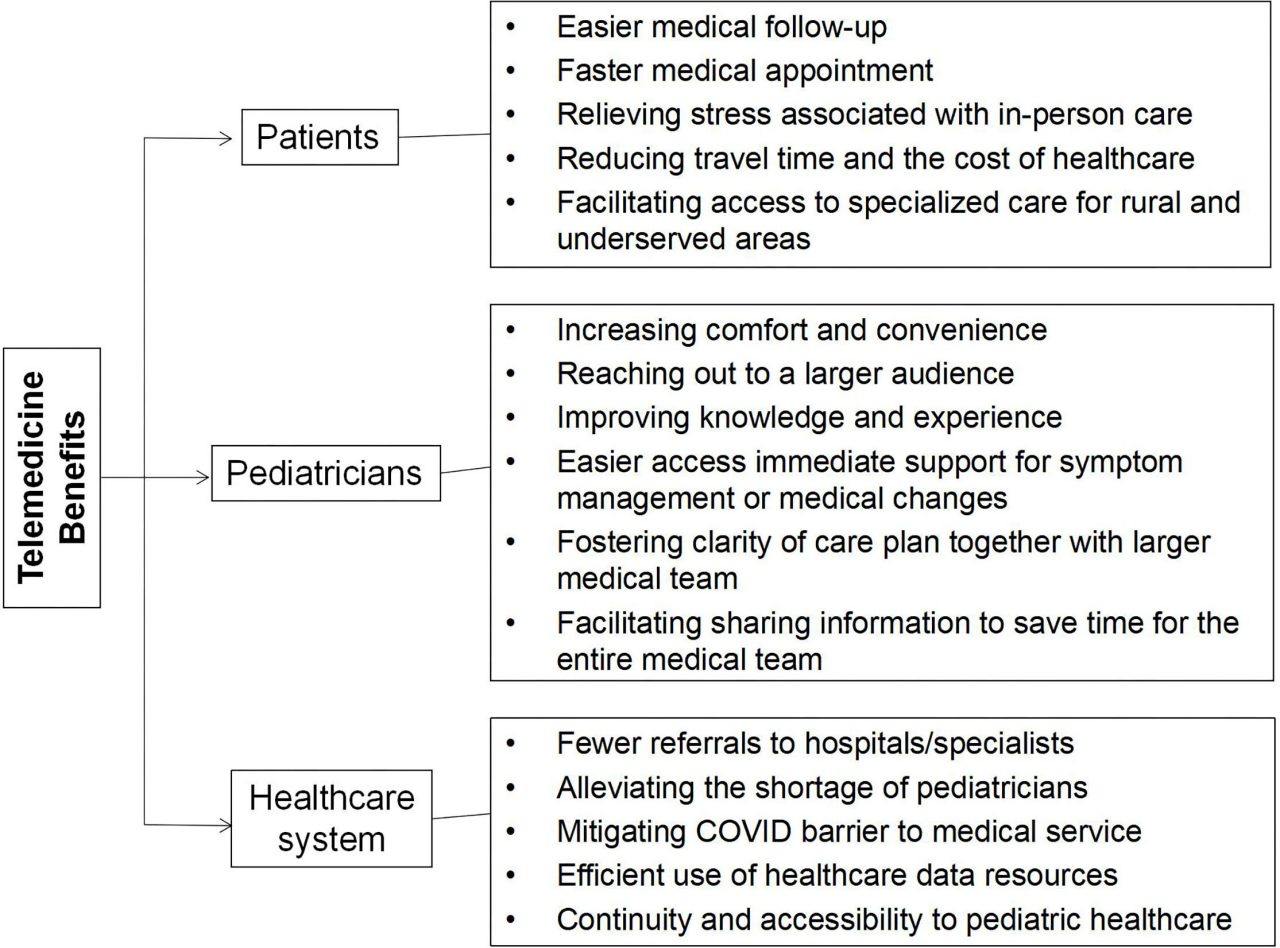
Telemedicine benefits for patients, pediatricians and healthcare system.

Price value was the strongest factor influencing telemedicine acceptance in a Chinese pediatric hospital. Higher-quality service should be provided relative to its perceived cost in order to price offerings appropriately to acquire and retain users. There is no doubt that now more than ever, is underscoring the importance of leveraging telemedicine service to optimize pediatric health care delivery in the current global COVID-19 pandemic. The finding of this study was to identify variables that may affect the adoption of telemedicine in order to determine actions and regulations that can be enacted to benefit all patients, healthcare providers and policymakers (Figure 2). Allied with relevant stakeholders in addressing ongoing and future challenges as well as cultural, logistical, technological, and financial barriers will be key for success. To guarantee the best experience possible for children, pediatricians, child patients, and their families, hospital administrators will have to take a leadership role in creating a standardized workflow, provide required technical support, and pay careful attention to integrating the training into workflows, enhancing service quality, durability, and user satisfaction of telemedicine services.

## Data Availability Statement

The raw data supporting the conclusions of this article will be made available by the authors, without undue reservation.

## Author Contributions

JS conceptualized the study, carried out the data analysis, drafted the initial manuscript, and reviewed and revised the manuscript. XY conceptualized the study, designed the data collection instrument, and supervised data collection. MW designed the data collection instrument, supervised data collection, and carried out the data analysis. PL conceptualized the study, designed the data collection instrument, carried out the data analysis, and reviewed and revised the manuscript. GY conceptualized the study, designed the data collection instrument, reviewed and revised the manuscript, and final approval of manuscript. All authors approved the final manuscript as submitted and agree to be accountable for all aspects of the work.

## Funding

This research was sponsored by National Natural Science Foundation of China (grant no: 72074146) and Science and Technology Commission of Shanghai Municipality-Shanghai Sailing Program (grant no: 21YF1451600).

## Conflict of Interest

The authors declare that the research was conducted in the absence of any commercial or financial relationships that could be construed as a potential conflict of interest.

## Publisher's Note

All claims expressed in this article are solely those of the authors and do not necessarily represent those of their affiliated organizations, or those of the publisher, the editors and the reviewers. Any product that may be evaluated in this article, or claim that may be made by its manufacturer, is not guaranteed or endorsed by the publisher.

## Abbreviations

AVE: average variance extracted
BI: behavioral intention
CR: composite reliability coefficient
EE: effort expectancy
FC: facilitating condition
H1: hypothesis 1
H2: hypothesis 2
H3: hypothesis 3
H4: hypothesis 4
H5: hypothesis 5
H6: hypothesis 6
HM: hedonic motivation
ICT: information and communication technology
ns: non-significant
PE: performance expectancy
PV: price value
UTAUT: unified theory of the acceptance and use of technology
UTAUT2: unified theory of the acceptance and use of technology 2
SD: standard deviation
SI: social influence.
