# Supplementary material for: Factors Influencing the Acceptance of Pediatric Telemedicine Services in China: A Cross-Sectional Study
Source: Front Pediatr. 2021 Oct 18;9:745687. doi: 10.3389/fped.2021.745687 (PMC8558490; doi:10.3389/fped.2021.745687)
Supplement: Supplementary file 1 [file Table_1.docx]

Table S1. Results of the questionnaire reliability analysis.

| Construct | Items | Cronbach α if item deleted | Cronbach α of the dimension |
| --- | --- | --- | --- |
| Performance Expectancy  (PE) | PE1 | 0.975 | 0.937 |
|  | PE2 | 0.975 |  |
|  | PE3 | 0.975 |  |
| Effort Expectancy  (EE) | EE1 | 0.975 | 0.957 |
|  | EE2 | 0.975 |  |
|  | EE3 | 0.975 |  |
| Social Influence  (SI) | SI1 | 0.975 | 0.914 |
|  | SI2 | 0.975 |  |
|  | SI3 | 0.976 |  |
| Facilitating Condition  (FC) | FC1 | 0.975 | 0.937 |
|  | FC2 | 0.974 |  |
|  | FC3 | 0.974 |  |
|  | FC4 | 0.975 |  |
| Hedonic Motivation  (HM) | HM1 | 0.975 | 0.907 |
|  | HM2 | 0.975 |  |
|  | HM3 | 0.977 |  |
| Price Value  (PV) | PV1 | 0.974 | 0.890 |
|  | PV2 | 0.975 |  |
|  | PV3 | 0.975 |  |
| Behavior Intention  (BI) | BI1 | 0.975 | 0.941 |
|  | BI2 | 0.975 |  |
|  | BI3 | 0.975 |  |
|  | BI4 | 0.975 |  |
