# Supplementary material for: Factors Influencing the Acceptance of Pediatric Telemedicine Services in China: A Cross-Sectional Study
Source: Front Pediatr. 2021 Oct 18;9:745687. doi: 10.3389/fped.2021.745687 (PMC8558490; doi:10.3389/fped.2021.745687)
Supplement: Supplementary file 2 [file Table_2.docx]

Table S2. Results of the questionnaire validity and confirmatory factor analysis.

| Construct | Items | Standardized factor loading | CR | AVE |
| --- | --- | --- | --- | --- |
| Performance Expectancy  (PE) | PE1 | 0.759 | 0.940 | 0.839 |
|  | PE2 | 0.783 |  |  |
|  | PE3 | 0.791 |  |  |
| Effort Expectancy  (EE) | EE1 | 0.840 | 0.958 | 0.884 |
|  | EE2 | 0.864 |  |  |
|  | EE3 | 0.875 |  |  |
| Social Influence  (SI) | SI1 | 0.847 | 0.924 | 0.803 |
|  | SI2 | 0.843 |  |  |
|  | SI3 | 0.754 |  |  |
| Facilitating Condition  (FC) | FC1 | 0.797 | 0.941 | 0.799 |
|  | FC2 | 0.892 |  |  |
|  | FC3 | 0.893 |  |  |
|  | FC4 | 0.860 |  |  |
| Hedonic Motivation  (HM) | HM1 | 0.813 | 0.907 | 0.766 |
|  | HM2 | 0.822 |  |  |
|  | HM3 | 0.577 |  |  |
| Price Value  (PV) | PV1 | 0.908 | 0.904 | 0.760 |
|  | PV2 | 0.799 |  |  |
|  | PV3 | 0.830 |  |  |
| Behavior Intention  (BI) | BI1 | 0.794 | 0.942 | 0.801 |
|  |  |  |  |  |
|  |  |  |  |  |
