# Supplementary material for: Factors Influencing the Acceptance of Pediatric Telemedicine Services in China: A Cross-Sectional Study
Source: Front Pediatr. 2021 Oct 18;9:745687. doi: 10.3389/fped.2021.745687 (PMC8558490; doi:10.3389/fped.2021.745687)
Supplement: Supplementary file 3 [file Data_Sheet_1.docx]

Supplementary Material

Summary of construct with measurement items.

| Construct | Serial number | Measurement items |
| --- | --- | --- |
| Performance  Expectancy (PE) | PE1 | I find telemedicine useful in my life. |
|  | PE2 | Using telemedicine helps me in managing my daily healthcare more quickly. |
|  | PE3 | Using telemedicine service increases my capability to manage my health. |
| Effort Expectancy (EE) | EE1 | Learning how to use telemedicine is easy for me. |
|  | EE2 | My interaction with telemedicine is clear and understandable. |
|  | EE3 | I find telemedicine easy to use. |
| Social Influence  (SI) | SI1 | People who are important to me think that I should use telemedicine services. |
|  | SI2 | People who influence my behavior think that I should use telemedicine. |
|  | SI3 | People in my society who use telemedicine service have more prestigious than those who do not. |
| Facilitating Condition  (FC) | FC1 | I have the resources necessary to use telemedicine services. |
|  | FC2 | I have the knowledge necessary to use telemedicine |
|  | FC3 | Telemedicine is compatible with other technologies I use. |
|  | FC4 | I can get help from others when I have difficulties using telemedicine services. |
| Hedonic Motivation (HM) | HM1 | Using telemedicine is fun. |
|  | HM2 | Using telemedicine is enjoyable. |
|  | HM3 | Using telemedicine is entertaining. |
| Price Value (PV) | PV1 | It enables me to use telemedicine services at a reasonable price. |
|  | PV2 | Telemedicine services is good value for the money. |
|  | PV3 | At the current price, telemedicine provides a good value |
| Behavior Intention (BI) | BI1 | I intend to accept medical services from telemedicine. |
|  | BI2 | I intend to order a telemedicine medical service in the future. |
|  | BI3 | I plan to receive health services from telemedicine frequently |
|  | BI4 | I will always try to use health service from telemedicine. |
